# Supplementary material for: Vaccination status and its determinants among children aged 12–23 months in Tigray, northern Ethiopia: A zero-inflated Poisson regression analysis
Source: PLoS One. 2025 Jul 28;20(7):e0327854. doi: 10.1371/journal.pone.0327854 (PMC12303341; doi:10.1371/journal.pone.0327854)
Supplement: S1 Checklist — (DOCX) [file pone.0327854.s001.docx]

STROBE Statement—Checklist of items that should be included in reports of **cross-sectional studies**

Study Title: **Vaccination status and its determinants among children aged 12-23 months in Tigray, northern Ethiopia: A** **zero-inflated Poisson regression analysis**

|  | Item No | Recommendation |
| --- | --- | --- |
| **Title and abstract** | 1 | (*a*) Indicate the study’s design with a commonly used term in the title or the abstract  In the abstract: page 2; line 44. |
|  |  | (*b*) Provide in the abstract an informative and balanced summary of what was done and what was found Abstract: Page 2; line from 44-62. |
| Introduction | | |
| Background/rationale | 2 | Explain the scientific background and rationale for the investigation being reported Introduction: pages 5 and 6; and line 111-125. |
| Objectives | 3 | State specific objectives, including any prespecified hypotheses Introduction: pages 5 and 6; line 123-125. |
| Methods | | |
| Study design | 4 | Present key elements of study design early in the paper Methods (on study design, setting sand period sub section) page 6; line 128. |
| Setting | 5 | Describe the setting, locations, and relevant dates, including periods of recruitment, exposure, follow-up, and data collection Methods (on study design and setting sub section) page 6; line 128-130. |
| Participants | 6 | (*a*) Give the eligibility criteria, and the sources and methods of selection of participants Methods (Study participants sub section) page 6; line 140. |
| Variables | 7 | Clearly define all outcomes, exposures, predictors, potential confounders, and effect modifiers. Give diagnostic criteria, if applicable Methods (Study variables and measurement sub section) page 7-9; line 163-191. |
| Data sources/measurement | 8* | For each variable of interest, give sources of data and details of methods of assessment (measurement). Describe comparability of assessment methods if there is more than one group Methods (**Data collection procedures and quality assurances**  **sub section) page** 7; line 156-161. |
| Bias | 9 | Describe any efforts to address potential sources of bias Methods (**Data collection procedures and quality assurances sub section) page** 7; line 152-161. |
| Study size | 10 | Explain how the study size was arrived at Methods (sampling procedure and sample size determination sub section) page 6; line 142-145. |
| Quantitative variables | 11 | Explain how quantitative variables were handled in the analyses. If applicable, describe which groupings were chosen and why Methods (data management and analysis sub section) page 9; line 196-197 |
| Statistical methods | 12 | (*a*) Describe all statistical methods, including those used to control for confounding Methods (data management and analysis sub section) page 9 and 10 ; line 197-208. |
|  |  | (*b*) Describe any methods used to examine subgroups and interactions |
|  |  | (*c*) Explain how missing data were addressed |
|  |  | (*d*) If applicable, describe analytical methods taking account of sampling strategy |
|  |  | (*e*) Describe any sensitivity analyses |
| Results | | |
| Participants | 13* | (a) Report numbers of individuals at each stage of study—eg numbers potentially eligible, examined for eligibility, confirmed eligible, included in the study, completing follow-up, and analysed Results page 10; line 219. |
|  |  | (b) Give reasons for non-participation at each stage |
|  |  | (c) Consider use of a flow diagram |
| Descriptive data | 14* | (a) Give characteristics of study participants (eg demographic, clinical, social) and information on exposures and potential confounders Results page 10-13; line 219-237. |
|  |  | (b) Indicate number of participants with missing data for each variable of interest |
| Outcome data | 15* | Report numbers of outcome events or summary measures Results page 13-15; line 239-250. |
| Main results | 16 | (*a*) Give unadjusted estimates and, if applicable, confounder-adjusted estimates and their precision (eg, 95% confidence interval). Make clear which confounders were adjusted for and why they were included. Results page 16-19; line 267-293. |
|  |  | (*b*) Report category boundaries when continuous variables were categorized |
|  |  | (*c*) If relevant, consider translating estimates of relative risk into absolute risk for a meaningful time period |
| Other analyses | 17 | Report other analyses done—eg analyses of subgroups and interactions, and sensitivity analyses |
| Discussion | | |
| Key results | 18 | Summarise key results with reference to study objectives Discussion page 19; line 295-302. |
| Limitations | 19 | Discuss limitations of the study, taking into account sources of potential bias or imprecision. Discuss both direction and magnitude of any potential bias Discussion (strength and limitation sub section) page 22; line 360-364. |
| Interpretation | 20 | Give a cautious overall interpretation of results considering objectives, limitations, multiplicity of analyses, results from similar studies, and other relevant evidence Conclusion page 22; lines 366-372. |
| Generalisability | 21 | Discuss the generalisability (external validity) of the study results bias Discussion 8(strength and limitation sub section) page 22; line 357-36. |
| Other information | | |
| Funding | 22 | Give the source of funding and the role of the funders for the present study and, if applicable, for the original study on which the present article is based  The study was financially supported by UNICEF, UNFPA, Amref Health Africa and Tigray Reginal Health Bureau. Its contents are solely the responsibility of the authors and do not necessarily represent the official views of the funders. The funders had no role in study design, data collection and analysis, decision to publish or preparation of the manuscript*.* |

*Give information separately for exposed and unexposed groups.

**Note:** An Explanation and Elaboration article discusses each checklist item and gives methodological background and published examples of transparent reporting. The STROBE checklist is best used in conjunction with this article (freely available on the Web sites of PLoS Medicine at http://www.plosmedicine.org/, Annals of Internal Medicine at http://www.annals.org/, and Epidemiology at http://www.epidem.com/). Information on the STROBE Initiative is available at www.strobe-statement.org.
