# Supplementary material for: Vaccination status and its determinants among children aged 12–23 months in Tigray, northern Ethiopia: A zero-inflated Poisson regression analysis
Source: PLoS One. 2025 Jul 28;20(7):e0327854. doi: 10.1371/journal.pone.0327854 (PMC12303341; doi:10.1371/journal.pone.0327854)
Supplement: Attachment — Submitted filename: Review comments.docx [file pone.0327854.s004.docx]

Review Comments:

The paper is well-constructed and addresses an important issue on childhood immunization, particularly in a conflict zone. However, addressing the following issues identified will make the study's results clearer to the reader:

1. Grammatical errors: the authors should read through for grammatical errors, particularly in lines 103, 128, 136, 249, and 390.
2. HHs is used for the first time on line 150. Kindly provide the full meaning at first mention.
3. Ethics: Why did you use verbal consent for mothers/caregivers ≥18 years but written assent for mothers/caregivers <18 years? Kindly put in a statement to explain the reason for this, which appears inconsistent.
4. Results: In Table 2, provide the full meaning of abbreviations such as ANC and PNC as footnotes. There are some inconsistent punctuations. E.g., 1,83 should be 1.83 (line 298), and 1,21-2,75 should be 1.27-2.75.
5. The authors had in their previous response indicated that they did not collect information on cultural barriers and other factors that could influence their conclusion. However, a statement about the potential influence of factors such as cultural and attitudinal barriers on vaccine uptake, even if they were not collected, will put their findings in context. This could be cited as a limitation of the study, a recommendation that future studies could factor.
