# Supplementary material for: Vaccination status and its determinants among children aged 12–23 months in Tigray, northern Ethiopia: A zero-inflated Poisson regression analysis
Source: PLoS One. 2025 Jul 28;20(7):e0327854. doi: 10.1371/journal.pone.0327854 (PMC12303341; doi:10.1371/journal.pone.0327854)
Supplement: Attachment — Submitted filename: Review comments.docx [file pone.0327854.s005.docx]

Review comments

PONE-D24-70501-R1

This study is a cross-sectional study assessing full, partial and no vaccination status for 12-23-year old children in Tigray, a region involved in conflict. (A few districts are excluded due to continuing security risks). Some demographic factors are analyzed as potential correlates of vaccination or lack thereof, including among others, education of the mother, media exposure, access to vaccination at local health facility, ante- and post-natal care. The data are described and analyzed using a Zero-Inflated Poisson (ZIP) method.

The paper is well-organized and clearly written. I am not sufficiently expert in statistical methods to evaluate the choice of modelling method, but it appears to be carefully considered and well justified.

Major comments:

Please clarify who the study participants are. Mothers of 12-23 month old children is the statement, but data seems to have been collected from 24-35 and so on (line 190-191). If the data on older children is to be analyzed later, please say so. Line 151-2 nearly says it. HOWEVER, note that on line 183 and following, and in the data, you discuss ‘delayed full vaccination’. Does this include only data up to month 23?

One section, Factors Associated with Child Vaccination Status, seems a bit repetitive and disorganized. I think it might be easier if a single factor was discussed at a time—its association with both full/partial and no-dose—and then the next factor rather than jumping back and forth. In particular, it is confusing to say that maternal education is associated with both full vaccination and no-vaccination status. It is technically true, but there is a positive association with full vaccination, and a negative association with no-vaccination. So is there clearer way to state all this? maternal education has a positive association with full vaccination, within the partial category does it alter # of vaccines?, and an educated mother is less likely to have a Zero-vaccinations child. Or perhaps just ignore the categories of full/partial/no vaccines and give the association: educated mothers get their children vaccinated more.

Uneducated should be defined; it seems to mean ‘no formal education’.

The ZIP method is described as a way to allow the extent to which a partially vaccinated child has received required vaccinations to be used, i.e. to bring into the evaluation whether partial vaccination is 1/14, 2/14…. Or 13/14 vaccines received (line 115-121). The data are presented in detail in Table 3. However, in the discussion, generally just the three categories, full, partial and no vaccination, are used.If the authors could make a brief statement regarding how the partial data influences their outcomes, it might help a relatively naïve reader such as myself.

The policy recommendations should be modified slightly, perhaps, to emphasize or clarify what could be done in the near future (radio or television promotion, encouragement to attend post-natal care, and especially provision of local vaccination services) vs what can be done long-term (improve education of women). Are there some steps that are more important in areas with poor media access or low education levels to compensate for these factors? One or two sentences might be helpful.

Minor comments:

Make sure that all abbreviations are written out and defined the first time they are used.

There are numerous small errors in the grammar. I will list some below and then in *italics* suggest a better way to phrase the statement.

Line 48 and elsewhere	‘was measured 14 antigens’	*was measured for 14 antigens*

Line 53 and elsewhere	‘ magnitude of fully vaccination’	*proportion of* *full vaccination	(or proportion that were fully vaccinated)*

Line 55 Being uneducated……….getting more vaccines among children. *The mother being educated (or having formal education) ……. (I’m not sure what is meant here—is it more vaccines per child, or likelihood of full vaccination?)*

*Please see comment above—lack of education is associated with children getting fewer vaccines, if I understand correctly)*

Line 76 mortality rate under five children *mortality rate of children under 5 years*

Line 77 Death of under 5 children.. *Death of children under (less than) 5 years old*

Line 83 Ethiopian providing routine	*Ethiopia provides routine*

Line 84 ten vaccines preventable	*ten vaccine-preventable*

Line 91 Despite the country	*Although the country*

Line 96 diseases outbreaks	*disease outbreaks*

Line 100 fist *first*

Line 102 was …compromised	*has been …..compromised*

Line 103-4 Only 16% of the health facilities in the region were provide immunization 103 services [18], and the

coverage of full vaccination was declined to 20.0% in 2021 [17]. *Only 16% of the health facilities in the region*

*were providing immunization services [18], and the coverage of full vaccination had declined to 20.0% in 2021 [17].*

Line 109 increase ….outbreaks. *increased…..outbreaks.*

Line 124 literatures	*literature*

Line 129 services nonetheless	*services, nonetheless*

Line 130	was limited *has been limited*

Line 130-131 assess child assess	*assess child vaccination*

Line 147 Study participants of the study	*study participants*

Line 157 literatures *literature*

Line 167 self-reports. *This might be a good place to describe briefly the measures you described in your response to reviewer to confirm self-reports.*

Line 173 Measles Cogitated Vaccine …. *Measles Containing Vaccine. (later in line 180 you just say Measles Vaccine. It would be good to be consistent.*

Line 184 12 years *12 months*

Line 191 35-49 years	*35-49 months*

Line 214 variables….was	*variables….were*

Line 217	ethical clearance….sought	*ethical clearance…..received (Sought is proper usage, but the requirement is to receive clearance not to seek it).*

Line 221 participants	*participants*

Line 221-2 if the participants were below the age of 18 years written informed assent was obtained…..

*If the participants (mothers) were below the age of 18 years, written informed CONSENT was obtained…?*

*‘assent’ is usually from children, whereas ‘consent’ is from adults, including for research involving their children. So I am guessing this was consent with ‘assent’ from the actual participant (teenage mother).*

Line 228-229 with nearly half ..of them were in the age group… *either say ‘with nearly half… of them in the age group..’ or ‘ years; nearly half….of them were in….’*

Line 229 three-fourth	*three-fourths*

Line 231	housewife *housewives*

Line 232	listened radio or watched television	*listened to radio or watched television	(not watched to television!) This has to be corrected in many places including Table 5.*

*Table 2. Abortion is not defined. It might not be so in Ethiopia, but in the US this is a very controversial issue and some would assume this to mean medically or surgically induced (which can be illegal), whereas it might mean spontaneous (‘miscarriage’) or both. You may or may not want to clarify. It seems irrelevant to this paper, and could be omitted.*

Line 249 were received	*had received*

Line 251 overdispersion *As a reader, I can’t interpret this. Perhaps its significance should be explained in the Discussion.*

Line 251 fully vaccination	*fully vaccinated*

Line 271 per child better…	*per child (omit better)*

Line 278-9 postnatal care (PNC) follow-up was significantly associated with zero-dose status among children aged 12-23 mo. *PNC followup had a significant negative association with zero-dose…..*

Line 280-281. 6% less likely …to get more vaccines…..born from educated….

*Born to educated mothers (also on line 290, 295)*

*I don’t understand what 6^ less likely to get ‘more vaccines’ means, mathematically. 6% sounds like it applies to a yes-no and ‘more vaccines’ sounds quantitative, like a slope. Same kind of question on line 282 and 286.*

Line 293 had not availability	*had no availability*

Line 296 had not receive PNC	*had not received PNC*

Line 317 …other studies conducted in Ethiopia (	)…… of children were completed

*…other studies of proportion of children completing all recommended vaccinations by one year of age, (Ethiopia, 55.9% [46],…..[49]).*

Line 318 was declined	*had declined from 73% prewar to 59.9% [14].*

Line 321 the same with	*the same as…Ethiopia as a whole*

*(is it all of Ethiopia? Or are you considering Ethiopia and Tigray as separate?.)*

Line 329 supported with	*supported by*

Line 336 had not availability	*had no availability	or	lacked availability*

Line 339-40 security concern	*security concerns*

Line 351 and 359 fully vaccination	*full vaccination*

*Is it true that media do promote vaccination? Is this a direct effect or an indirect effect, or do you know?*

Line 357	were high chance	*were at high risk*

Line 364	Strength and limitation	*Strengths and limitations*

*(Suggest clearly separating this into strengths and limitations. Strengths of this study are … Is the ZIP method novel enough to be a strength? Limitations of the study include that, in case the child immunization….*

*Figures need to be numbered.*

*On Figure 3, ‘not knowing to back …’ is not very clear. Perhaps not knowing to return??*
