# Supplementary material for: Vaccination status and its determinants among children aged 12–23 months in Tigray, northern Ethiopia: A zero-inflated Poisson regression analysis
Source: PLoS One. 2025 Jul 28;20(7):e0327854. doi: 10.1371/journal.pone.0327854 (PMC12303341; doi:10.1371/journal.pone.0327854)
Supplement: Attachment — Submitted filename: Review of PONE-D-24-50701R1.docx [file pone.0327854.s006.docx]

Some of the concerns were already corrected that have been pointed out by another excellent reviewer.

As for the data collected, I believe that it has been sufficiently examined in a situation where the literacy rate is low and in the context of conflict.

The main result is that the fully vaccinated rate is 59.9%, largely due to vaccine shortages (47.0%), security concerns (12.3%), health facility closures (11.0%) because of the conflict, and that the vaccination rate of children of ill-informed mothers is low. We believe that this study will provide basic data for improving the vaccination rate in Ethiopia.

As an aside, in times when the literacy rate was not high in my country, songs that were popular among the common people sometimes led the country in a good direction. I hope you find it helpful.

I suggest that the Editor deserves to be published.
